# Contact Transfer Epitaxy of Halide Perovskites

**DOI:** 10.1002/adma.202308892

**Published:** 2025-07-09

**Authors:** Hongyu Sun, Linde M. van de Ven, Benjamin Duncan, Eva Stein, Daan Methorst, Maryam Mohammadi, Susan A. Rigter, Peter Schall, Erik C. Garnett

**Affiliations:** ^1^ AMOLF Science Park 104 Amsterdam 1098 XG The Netherlands; ^2^ University of Amsterdam Science Park 904 Amsterdam 1098 XH The Netherlands

**Keywords:** EBSD, epitaxy, nanocubes, perovskites

## Abstract

Halide perovskite materials are very exciting because of their excellent optoelectronic properties and simple deposition with both solution and vapor‐phase methods. Until now, solution deposition has received more attention, but there are growing indications that residual solvent may limit performance and in particular long‐term stability. Evaporation is a promising alternative, but is more complicated to implement; it requires vacuum, multiple sources at different temperatures, is difficult to switch between different materials due to cross‐contamination and typically leads to films with very small grain size. Here a novel contact transfer method is presented for fabricating single crystalline perovskites that maintains the simplicity and flexibility of solution deposition while avoiding the use of solvent. This contact transfer epitaxy method uses an acceptor substrate consisting of self‐assembled perovskite nanocubes to control crystal orientation and a donor substrate of the desired perovskite film to determine the ultimate composition. By heating the two substrates under close contact in atmospheric conditions, the perovskite film is transferred from the donor to the acceptor substrate, showing cubic phase (100) orientation even with hexagonal donor films. It is shown that contact transfer epitaxy is compatible with a variety of compositions and does not require specialized evaporators or vacuum conditions.

## Graphical abstract

Bringing a halide perovskite thin film (donor) into contact with a self‐assembled monolayer of oriented perovskite nanocrystals (acceptor) at elevated temperature leads to epitaxial transfer of halide perovskite from the donor to the acceptor substrate. The acceptor substrate controls the crystal orientation and grain size, while the donor substrate controls the composition.

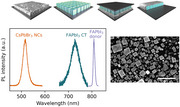

## 1 Introduction

Record photovoltaics (PVs) and light‐emitting diodes (LEDs) consist of single crystalline semiconductors because of their superior optoelectronic quality compared to polycrystalline materials.^[^ ^1^, ^2^, ^3^ ^]^ In halide perovskite materials, there is still a debate over the role of grain boundaries,^[^ ^4^ ^]^ but many of the lowest threshold lasers and lowest trap densities are measured in single crystals.^[^ ^5^, ^6^, ^7^, ^8^ ^]^ This has stimulated a large body of research devoted to methods for fabricating single crystalline perovskite thin films.^[^ ^9^, ^10^, ^11^, ^12^ ^]^ Many of these approaches use solution‐based deposition, which limits the compatibility of underlying layers, requires specific wetting conditions and can leave behind residual solvent that may reduce long term stability and performance.^[^ ^13^, ^14^, ^15^ ^]^ Thermal evaporation relaxes the restriction of substrate compatibility, and can provide uniform films, but typically leads to polycrystalline layers, requires high vacuum conditions and needs multiple sources at very different temperatures.^[^ ^16^, ^17^, ^18^, ^19^ ^]^ Vapor phase epitaxy has been achieved, but requires high vacuum and lattice matched substrates, which limits compatibility with typical contact layers.^[^ ^20^, ^21^, ^22^ ^]^ Here we present a new contact transfer epitaxy method that allows us to deposit single crystalline perovskite layers with a variety of compositions on arbitrary substrates. We do this by using an acceptor substrate to control film crystallinity and a donor substrate to determine film composition. The acceptor substrate consists of a self‐assembled layer of perovskite nanocubes, which templates the crystal orientation and phase. The donor substrate is a perovskite film with any desired composition. By heating the acceptor and donor substrates under close contact in atmospheric conditions, we can transfer the perovskite film from the donor to the acceptor substrate. The heating can be provided by a simple tube furnace, avoiding the use of specialized evaporators and eliminating the need for high vacuum. We show that the method is compatible with a variety of compositions including but not limited to CH_3_NH_3_PbI_3_ (MAPbI_3_), CH(NH_2_)_2_PbI_3_ (FAPbI_3_), CsSnBr_3_, FASnI_3_, and MASnI_3_. We use x‐ray diffraction (XRD), electron backscatter diffraction (EBSD), absorption, photoluminescence (PL), and energy‐dispersive X‐ray spectroscopy (EDS) to confirm the crystal phase, orientation, and optical properties of the final films.

## 2 Results

**Figure 1:**
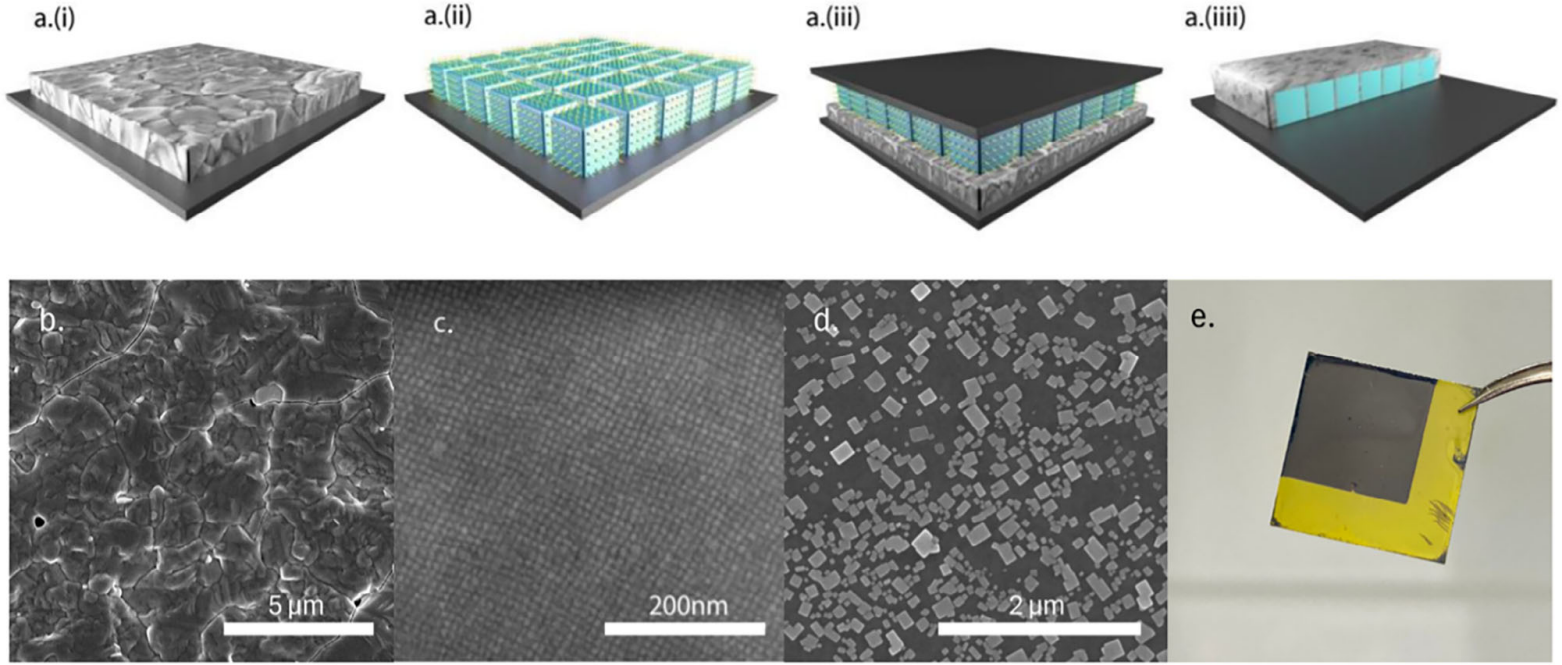
a) Schematic diagram of the contact transfer process. The SEM images show b) the FAPbI_3_ donor film before contact transfer, c) the self‐assembled CsPbBr_3_ nanocube acceptor layer, d) the highly oriented overgrown crystals formed after contact transfer. e) shows the donor film after performing contact transfer with MAPbI_3_ in air on a 12×12 mm substrate. The dark area was covered by the acceptor substrate, whereas the yellow area was exposed to air.

The contact transfer epitaxy process is shown in **Figure** **1**. We start with two components: a spin‐coated donor perovskite film and a self‐assembled CsPbBr_3_ perovskite nanocube acceptor layer. These are shown in Figure 1a(i) and Figure 1a(ii), respectively. The details of the spin coating and self‐assembly procedures are described in the Experimental Section. Figure 1b,c depicts the corresponding SEM images of the donor and the acceptor layers. We place the two layers in contact with each other and transfer them into a tube furnace that is already at the setpoint temperature (180–220 °C, depending on donor composition) for accurate control of heating temperature and time. After this process, we found that the perovskite is transferred from the donor to the acceptor film.

Figure 1d shows an example of the result after contact transfer of FAPbI_3_. Square shaped crystals with dimensions varying from 100 to 500 nm are observed on the acceptor substrate. The crystals are all aligned in the same direction due to the nanocube assembly. In order to confirm the effects of the donor and acceptor layer, we perform two reference experiments: the same process but without the acceptor layer, and the same process without the donor layer. When the acceptor layer is left out, no perovskite transfer is observed from the donor to the acceptor substrate, and the result is a bare silicon substrate, and when the donor layer is left out, only minor changes are observed to the layer of nanocrystals (Figure S1, Supporting Information). Only when the two layers come in contact, we observe the beautifully aligned crystals on the acceptor substrate after the transfer process.

An additional advantage of the technique is that we found that covering the acceptor film with the donor substrate can prevent thermal degradation of the donor perovskite. When performing contact transfer with MAPbI_3_ in air at 180 °C, two areas can be observed on the donor substrate (Figure 1e). We can see the area not covered by the acceptor substrate turns yellow quickly when the temperature reaches 180 °C, indicating that PbI_2_ forms, while the area covered by the acceptor substrate remains black, revealing the presence of the right MAPbI_3_ phase. This is further confirmed by XRD measurements of both the black and yellow phase (Figure S2, Supporting Information). The reason for this is that the covering protects the film from coming into contact with oxygen in the atmosphere and the close confinement also traps any decomposition products, promoting back reaction to form the perovskite. Hence, in contrast to thermal evaporation, contact transfer epitaxy does not require vacuum conditions or multiple sources with different temperatures, making it easier to implement.

**Figure 2:**
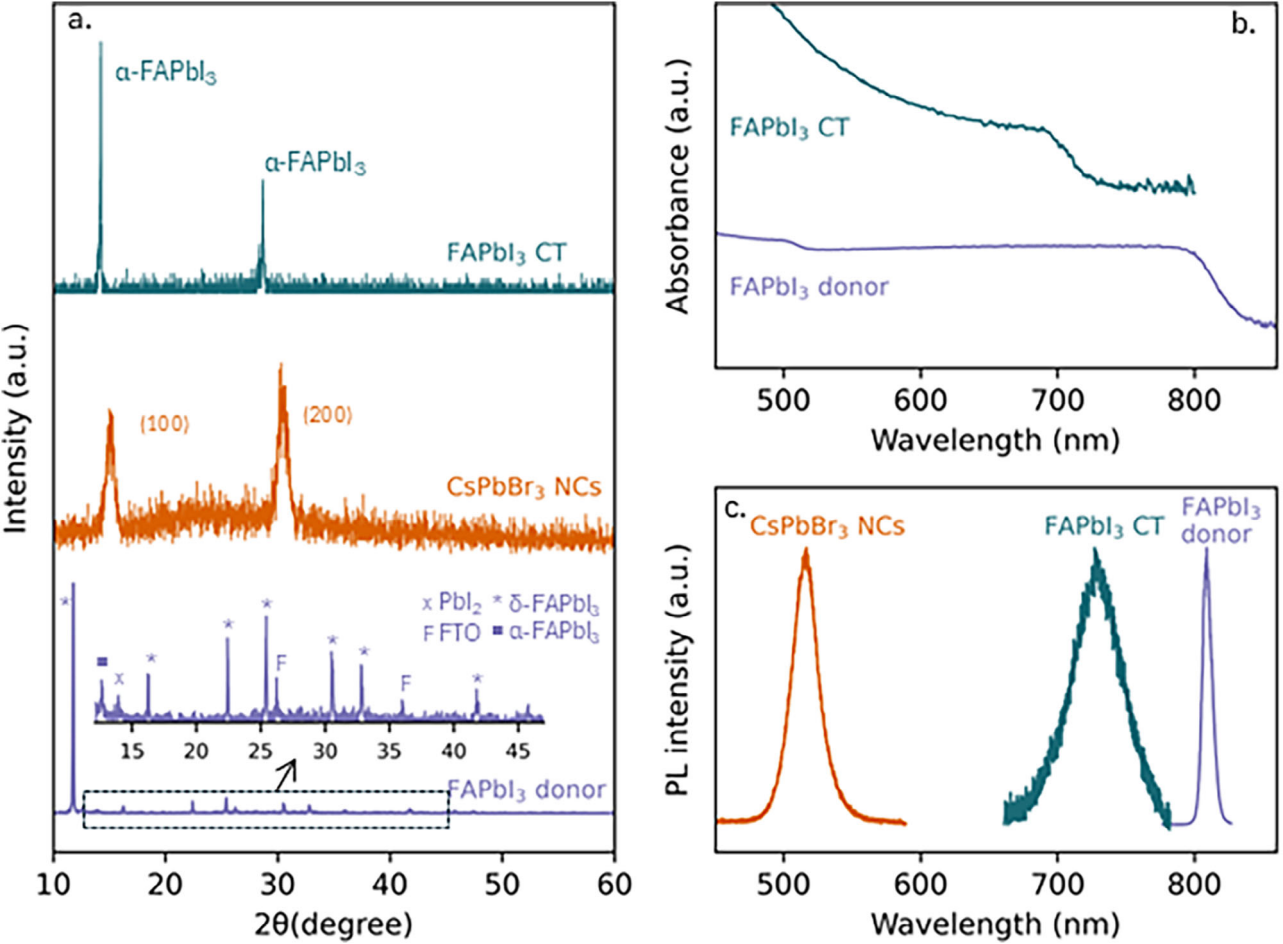
Contact transfer of FAPbI_3_. a) XRD patterns of FAPbI_3_ crystals from contact transfer (CT), CsPbBr_3_ self‐assembled nanocube layer (NCs), and FAPbI_3_ donor film, with the peaks from each material labeled, b) UV–vis absorption spectra, c) PL spectra.

To characterize the transferred crystals, we have performed XRD, optical absorption, and PL measurements on the acceptor layer after contact transfer. Contact transfer with organic cation perovskite FAPbI_3_ is shown in **Figure** **2**a (sample after contract transfer named CT, the same naming abbreviation for other materials used in this report). The XRD shows that the donor film contains a strong hexagonal δ phase FAPbI_3_ component in addition to the cubic phase. The film is thus dominated by its hexagonal phase that is undesirable for solar cell applications.^[^ ^23^ ^]^ Notably, we also observed a trace amount of PbI_2_, which is common in solution‐processed FAPbI_3_ films.^[^ ^24^, ^25^ ^]^ Despite the relatively low fraction of the cubic phase, we still detected PL signal from the donor film which is shown in Figure 2c. Surprisingly, the acceptor substrate after contact transfer epitaxy only shows the desired α phase of FAPbI_3_. This is evidence that the cubic phase CsPbBr_3_ acceptor layer acts as a growth promotor, templating the epitaxial crystallization of FAPbI_3_ during contact transfer.

The PL spectrum in Figure 2c shows that the PL peak from the contact‐transferred FAPbI_3_ film is shifted with respect to that of the original FAPbI_3_ donor film, toward that of the CsPbBr_3_ nanocubes. In addition, we observe an absorption onset between 500 and 600 nm (Figure 2b), which is consistent with a wider bandgap perovskite compared to pure FAPbI_3_. At the same time, the absorption onset curve is also less steep than FAPbI_3_ from other works.^[^ ^26^, ^27^ ^]^ Taken together, all these results indicate that there is halide mixing at the interface between the donor and acceptor after contact transfer epitaxy. Such mixing is unsurprising given the rapid interdiffusion of halides that can occur even at room temperature.^[^ ^28^ ^]^

A contact transfer temperature of 200 °C is high enough to evolve FAI from FAPbI_3_.^[^ ^29^ ^]^ FAI vapor from the decomposed perovskite donor substrate could convert CsPbBr_3_ to a mixed organic–inorganic FA_x_Cs_1−x_PbI_y_Br_3−y_ phase. To further confirm that our contact transfer technique transfers the perovskite composition stoichiometrically and does not just convert CsPbBr_3_ we use a donor substrate containing fully inorganic CsSn_2_Br_5_ perovskite.

Using the inorganic Sn‐based perovskite has several benefits. First, due to its purely inorganic nature, the decomposition temperature is substantially higher; MAPbI_3_ and FAPbI_3_ start to decompose below 300 °C,^[^ ^30^ ^]^ while CsSnBr_3_ is stable above 400 °C.^[^ ^31^ ^]^ Therefore, using the fully inorganic perovskites allows us to assess the applicability of the contact transfer method to materials that are more thermally stable. Second, the absence of Pb in the donor film allows us to prove that B‐site cations are transferred with our method. Third, the CsSn_2_Br_5_ perovskite we used forms a hexagonal phase on the donor substrate and thus we can test if the cubic CsPbBr_3_ nanocubes template the crystallization and control the phase even when no cubic phase is present at all in the donor film.

**Figure 3:**
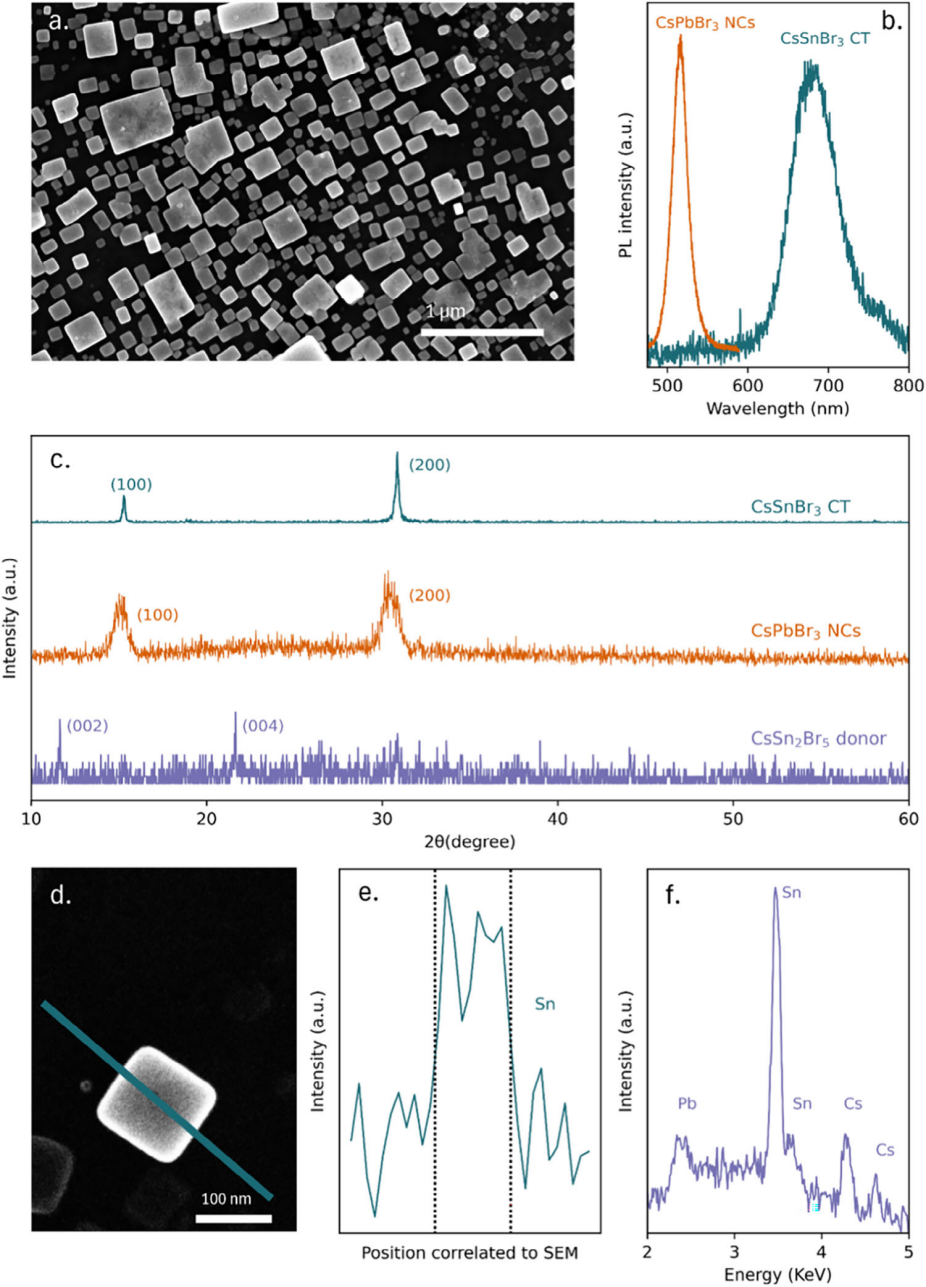
Contact transfer of CsSn_2_Br_5_. a) SEM image of the overgrown CsSnBr_3_ crystals from contract transfer epitaxy, b) PL spectra of contact transfer and CsPbBr_3_ self‐assembled nanocube acceptor layer, c) XRD patterns of contact transfer, acceptor layer and CsSn_2_Br_5_ donor film d) SEM image of the crystal used for EDS and e) the Sn element linescan, with the dashed lines corresponding to the crystal edges in the SEM image, f) EDS spectrum from the same crystal accompanied by Cs Lα and Lβ peaks at 4.286 and 4.848 keV, respectively.

**Figure** **3**a shows the SEM image of the acceptor substrate after contact transfer epitaxy with CsSn_2_Br_5_. It displays an identical cubic morphology as observed previously with the FAPbI_3_ donor film. This suggests that CsPbBr_3_ nanocubes act as an epitaxial substrate, templating the crystallization of the transferred film to form a highly oriented cubic crystal phase. Figure 3b shows the material has a broad PL peak at 690 nm, which is red‐shifted from the CsPbBr_3_ emission but consistent with cubic CsSnBr_3_ or mixed CsPb_x_Sn_1‐x_Br_3_ perovskite.^[^ ^32^, ^33^, ^34^, ^35^ ^]^ Mixed PbSn perovskite has a bandgap similar or even redshifted compared to Pb perovskite,^[^ ^35^ ^]^ making it difficult to distinguish between the different perovskites without knowing the exact composition.

The XRD pattern in Figure 3c confirms that contact transfer converted the original hexagonal CsSn_2_Br_5_ donor film into cubic CsSnBr_3_. Compared to the original CsPbBr_3_ nanocubes on the acceptor substrate, the contact transfer substrate shows a shift in XRD peak position, increase in intensity and narrowing of the full width half maximum, all consistent with epitaxial growth of Sn perovskite on the nanocube template.

To further verify the transfer of material we used energy dispersive spectroscopy (EDS). The linescan across one crystal (Figure 3d,e) shows strong Sn signals that are spatially localized to the cubic structures. Unless specified otherwise, all EDS scanning data was collected for 5 min under 20 kV electron accelerating voltage to avoid film damage from long exposure time. The energy spectrum in Figure 3f shows the presence of Sn, Cs, Pb, and Br in the material, as expected. From these observations, we can state that cubic phase CsPbBr_3_ nanocubes promote the conversion of hexagonal CsSn_2_Br_5_ to cubic CsSnBr_3_. This phase stabilization phenomenon is also seen in another report of perovskite epitaxy.^[^ ^22^ ^]^ Our results demonstrate that inorganic ions in perovskite films can be transferred over a short distance to the acceptor substrate, even when the temperature is far below the melting point of the film (437 °C).^[^ ^36^ ^]^

Contact transfer is possible with different donor compositions. In the Supplementary data we demonstrate contact transfer with MAPbI_3_ (Figure S3, Supporting Information), FASnI_3_ (Figure S4, Supporting Information), and FASnBr_3_ (Figure S5, Supporting Information).

**Figure 4:**
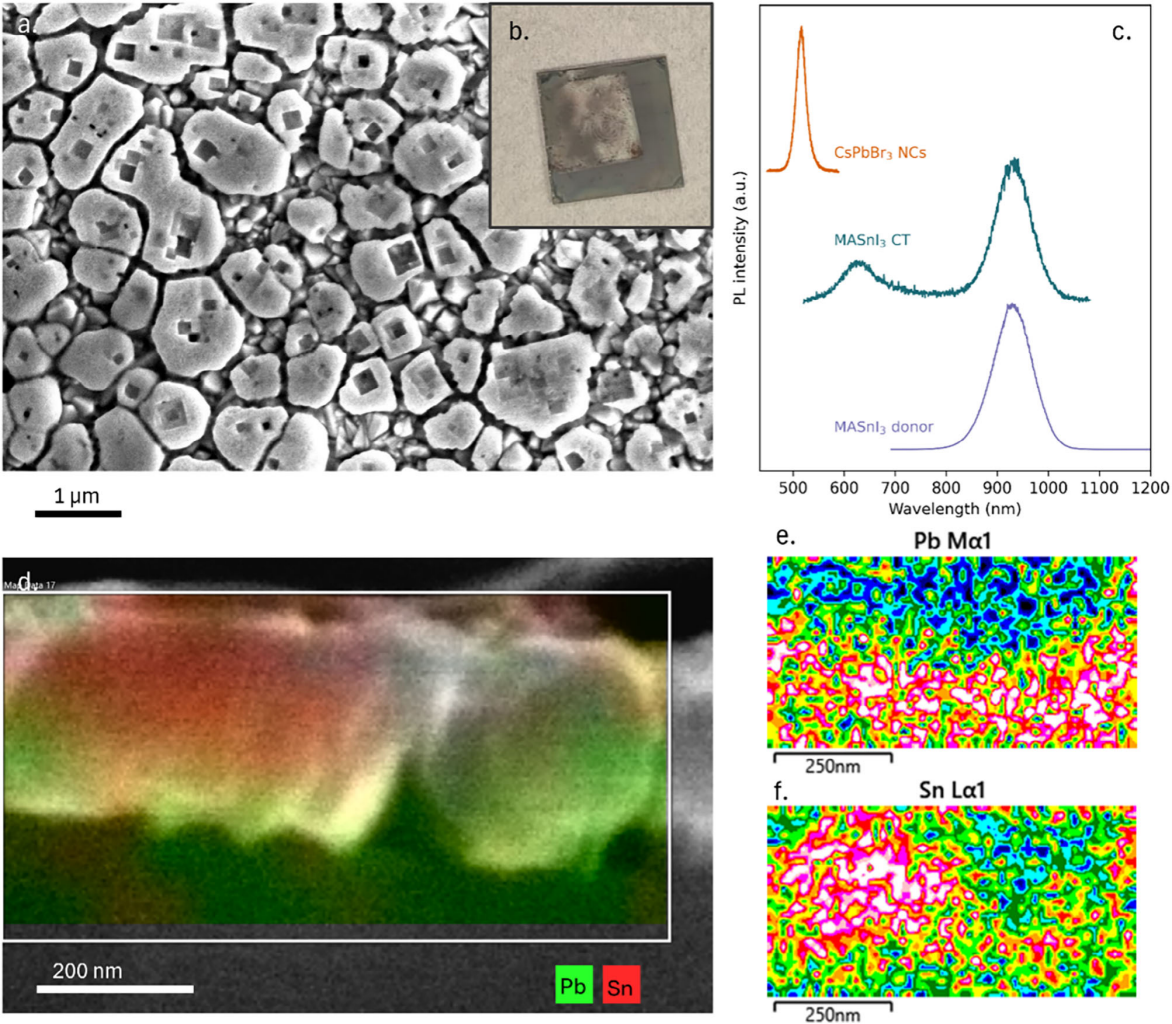
Contact transfer of MASnI_3_ at elevated temperature. a) SEM picture of MASnI_3_ donor with cubic gaps, b) photograph of 12×12 mm MASnI_3_ donor substrate with transparent region of contact transfer, and c) PL spectra of CT, donor and acceptor. d) EDS map of cross‐section of MASnI_3_ CT. Sn and Pb maps are shown individually in (e) and (f), respectfully.

Contact transfer with MASnI_3_ is performed at a slightly elevated temperature (220 °C instead of 200 °C). An SEM picture of the donor film (**Figure** **4**a) shows cubic “gaps” where material transferred from the donor to the acceptor film. These gaps are not visible on the donor film outside the contact transfer region (Figure S6a, Supporting Information). This indicates that a substantial amount of material transfers from the donor substrate to the acceptor substrate. This can also be seen by eye in Figure 4b, which shows a photograph of the donor substrate after contact transfer. The more transparent area is the area that was covered by the acceptor substrate, while the gray region was not.

Figure 4c shows a double PL peak coming from the acceptor substrate after contact transfer epitaxy. The emission peak centering around 620 nm around stems from a mixed MA_x_Cs_1−x_Pb_y_Sn_y‐1_I_z_Br_3−z_ perovskite, and the peak around 950 nm can be attributed to pure MASnI_3_ perovskite emission.^[^ ^37^ ^]^ This peak splitting suggests that first pure MASnI_3_ grows epitaxially on the CsPbBr_3_ nanocube acceptor substrate followed by interfacial interdiffusion of iodide and bromide. This can be further confirmed by XRD measurements, where at 15 and 30° double peaks appear (Figure S6c, Supporting Information). The slightly lower angle peaks match the expected position of the MASnI_3_ donor (001) and (002) planes, respectively,^[^ ^37^ ^]^ while the higher angle peaks correspond to the CsPbBr_3_ (100) and (200) planes.

It could be expected that at 220 °C organic cation MAI could decompose or escape from the edges of the two substrates in the form of MAI vapor. However, the PL peak around 950 nm (Figure 4c) cannot be explained as a result of mixing of only Sn and I ions into the acceptor layer.

In the case of complete replacement of Pb and Br with Sn and I, respectively, to form CsSnI_3_, the emission would be expected at 920 nm.^[^ ^38^ ^]^ In contrast, after contact transfer epitaxy, we observe a more redshifted peak that overlaps perfectly with the emission of the donor perovskite film MASnI_3_. Furthermore, if the organic cation would decompose and not transfer to the acceptor, we would expect decomposition products like PbI_2_ and SnI_2_ to be present in the XRD pattern (Figure S6c, Supporting Information), as there would not be a stochiometric ratio of perovskite components. We speculate that the close contact between the donor and acceptor substrates leads to mass transfer inhibition, which prevents most of the organic species from leaving, enabling stoichiometric transfer from the donor to the acceptor substrate.

To elucidate the nature of the interface between the donor and acceptor substrate, we look at cross‐sectional EDS mapping of MASnI_3_ contact transfer (Figure 4d). This data shows substantial halide mixing throughout the cross‐section of the material, as well as even distribution of the Cs cation that is only present in the acceptor layer (Figure S7, Supporting Information). We can conclude that there is ionic interdiffusion. This can also explain the presence of mixed perovskite emission in Figures 2, 3, and 4. However, a clear distinction between the less‐mobile Pb and Sn ions is visible in Figure 4d–,f. A layer of Pb enrichment can be seen close to the substrate, whereas more Sn is present at the top of the sample.

**Figure 5:**
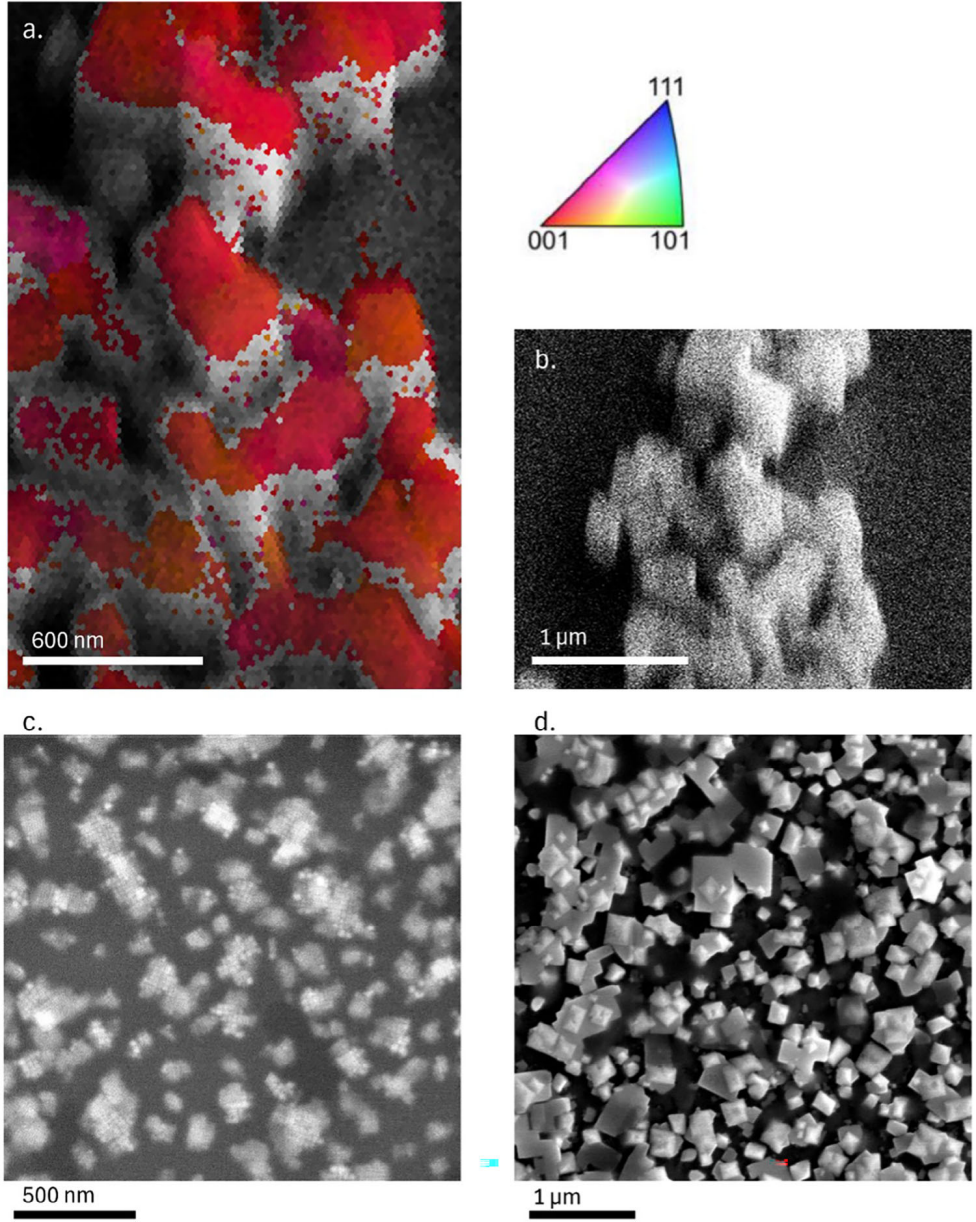
a) EBSD orientation map of CsSnBr_3_ crystals formed by contact transfer epitaxy, with the color legend in the top right corner indicating the orientation, b) SEM of the same area. c) SEM of acceptor substrate with smaller regions of self‐assembled nanocubes, d) SEM of MASnI_3_ CT using (c) as an acceptor substrate.

To show that the transfer of material is indeed epitaxial, we study the orientation with nanoscale spatial mapping using electron backscatter diffraction (EBSD). This method is used to investigate crystal grain size and orientation for a wide range of materials such as metals, minerals and ceramics.^[^ ^36^ ^]^ Recently, EBSD has also been applied to electron beam sensitive materials like perovskites by carefully controlling the electron dose.^[^ ^39^, ^40^, ^41^, ^42^ ^]^ Here (**Figure** **5**), we use this technique to study the crystal orientation after contact transfer with CsSn_2_Br_5_. The optimized scanning parameters of 8 kV, 100pA, and an integration time of 120 ns give a clear Kikuchi pattern without damaging our CsSnBr_3_ sample (two examples of the Kikuchi patterns are included in Figure S8a, Supporting Information to show the pattern quality). By removing the non‐indexable points from scanning, we fitted the remaining points to a cubic phase CsSnBr_3_ model, leading to the final indexed EBSD map shown in Figure 5a. Every color point corresponds to the crystal orientation at that specific location, with the color legend shown in the figure inset. Because of roughness that can be seen in the SEM image (Figure 5b), only the top surface of the crystals can give EBSD signals. This is common in EBSD, because the samples are tilted at 70°, such that any diffracted electrons coming from the side of the crystal cannot arrive correctly at the detector. Nevertheless, all distinguishable crystals show (001) orientation; this result is consistent with the orientation we found in XRD. The nanoscale mapping, however, allows us to locally define the mis‐orientation angle within one single grain.^[^ ^40^, ^43^ ^]^ From two example linescans (Figure S8b, Supporting Information) we can see only 2° and 4° mis‐orientation angles, which indicates the overgrown layer is in a controlled orientation templated by the nanocube assembly.

To demonstrate that the orientation of the material is dependent on the orientation of the nanocube layer underneath, we performed contact transfer on a substrate that has nanocubes aligned only in very small regions (Figure 5c). When using this acceptor substrate, we expect regions to grow epitaxially, but for the different crystals to no longer all have the same orientation. After contact transfer, we indeed observe epitaxially grown crystals, but there is no long range order (Figure 5d). This shows the epitaxial nature of the perovskite transfer from the donor substrate to the acceptor substrate.

We characterized the photovoltaic properties of the donor and acceptor film using spatially resolved photoluminescence quantum yield (PLQY) and implied open circuit voltage (iV_OC_) measurements (Figure S9, Supporting Information). Such measurements are often preferred over full device efficiency when screening new process steps, since they are directly related to the intrinsic improvements in the quality of the material or interface and can be probed without re‐optimizing the entire device fabrication process.^[^ ^44^ ^]^ The PLQY measurements performed on the acceptor substrate (Figure S9a, Supporting Information) show sub‐micron sized regions with high quantum yield. The size of these regions corresponds to the size of the epitaxially grown perovskite crystals (Figure 1d, Figure 3c). These regions are surrounded by regions with PLQY<1%, where there is no perovskite present on the substrate. The donor film (Figure S9b, Supporting Information) has a more uniform distribution of PLQY values, with a slightly lower overall PLQY compared to the acceptor substrate. The same regions show a lower iV_OC_ for the donor substrate than for the acceptor substrate (Figure S9c,d, Supporting Information), which can be partly attributed to the difference in bandgap between the acceptor and the donor. The high PLQY and iV_OC_ of the acceptor substrate show the potential of this method for use as an active layer in PV or LED devices.^[^ ^44^ ^]^

## 3 Discussion

The contact transfer epitaxy process demonstrates that perovskite can undergo thermal overgrowth where the composition can be determined by a donor substrate and the crystallinity dictated by the acceptor substrate. The process only occurs when both donor and acceptor films are present; Figure S1 (Supporting Information) shows that in the absence of either the acceptor nanocube layer or the donor perovskite film no transfer occurs. The crystal phase of the transferred layer is derived from the acceptor layer, as demonstrated by XRD and SEM results presented in Figures 2, 3, and 4, which exhibit a cubic phase and square crystal shape characteristic of the nanocube acceptor layer. The orientation of the transferred perovskite crystals is dictated by the orientation of the self‐assembled nanocube acceptor film, as shown in Figure 5, while the composition is controlled by the donor film, as evidenced by the EDS results presented in Figure 4.

All of this taken together strongly suggests an epitaxial growth mechanism, but we acknowledge that the low decomposition temperature and high atomic mobility of halide perovskites complicate this assignment. In traditional epitaxy, the growth substrate has a fixed lattice that templates the crystallinity of the overgrown layer. In our case, the mixed composition observed after contact transfer suggests that the epitaxial template itself is changing during overgrowth, although we cannot rule out the possibility that traditional epitaxy occurs followed by interdiffusion of ions. Although there are some differences with traditional epitaxy, we still call this process contact transfer epitaxy because of the dominant (001) orientation and crystalline phase locking displayed in the transferred layer. This is in line with terminology used in other non‐traditional epitaxy, such as van der Waals epitaxy.^[^ ^45^ ^]^

One area where the method still needs improvement is in making compact thin‐film layers. We see that despite having a close‐packed layer of nanocubes on the acceptor substrate and a complete thin‐film on the donor substrate, after contact transfer epitaxy the acceptor substrate consists of single crystalline islands with some nanoscale gaps between them, rather than a continuous thin film. We expect that this arises due to ligand removal and merging of the CsPbBr_3_ nanocube layer during the contact transfer epitaxy process. When heated, the nanocubes merge together, and create islands on which the epitaxial layer grows. We see only minor agglomeration during heating without the presence of the donor film (Figure S1, Supporting Information), so it seems both heat and additional perovskite is critical to strip the ligands. We expect that more rapid heating protocols for the donor substrate, using for example light or current pulses, may solve this problem, which is the focus of current investigations.

Although island‐like perovskite is not suitable for use in high efficiency solar cells, recent publications have shown that morphology similar to what we observed with contact transfer epitaxy might be ideal for use in LEDs.^[^ ^46^, ^47^, ^48^, ^49^ ^]^ This is because the island morphology can induce higher PLQY, better light outcoupling efficiency, and higher stability in LEDs.^[^ ^46^, ^47^, ^48^, ^49^ ^]^ Lower charge carrier mobility and more confined carriers lead to higher radiative recombination rates, and the island morphology leads to enhanced light outcoupling.^[^ ^47^, ^48^ ^]^ Shunt current pathways that are detrimental for solar cells can be easily prevented in LEDs by adding an organic insulating layer on top of the perovskite layer.^[^ ^47^, ^48^ ^]^ For solar cells, such barrier layers at the contacts that are necessary to prevent shunting reduce extracted current, while the gaps in between islands lower absorption of incoming sunlight, again lowering photocurrent and efficiency.^[^ ^50^ ^]^ Although this island morphology is not suitable for high efficiency solar cells, there has been some success in applying it for semi‐transparent halide perovskite solar cells.^[^ ^50^ ^]^

## 4 Conclusion

We have developed a method to transfer a wide variety of perovskite compositions from a donor film substrate to an acceptor film substrate containing a self‐assembled nanocube film.

The process requires close contact between the donor and acceptor substrate, which protects the perovskite materials and allows for the use of a simple oven, removing the need for a specialized evaporator. The transferred film crystal phase and orientation is controlled by the acceptor substrate, adopting its cubic (100) preferred orientation, while the composition is controlled by the donor substrate. Our contact transfer process transforms mixed (hexagonal and cubic) phase FA/MASn/PbBr/I_3_ into pure cubic phase, while completely converting hexagonal Sn‐based perovskite into the cubic phase. The EBSD results show a uniform orientation in the transferred crystals with less than 5° misorientation. Photoluminescence and absorption measurements confirm that stoichiometric perovskite donor film transfer occurs, though shifts in the bandgap indicate some alloying with the acceptor nanocube film. The ability to deposit perovskites with controlled composition, crystal phase and orientation without solvent using a simple oven opens up interesting processing conditions that could be relevant for industrial applications of halide perovskite optoelectronics.

## 5 Experimental Section

### Materials

*CsPbBr_3_ Nanocubes*: Cesium carbonate (Cs_2_CO_3_, >99.99%), lead(II) bromide (PbBr_2_, >99,99%), oleylamine (OAm, >98%), oleic acid (OA,>90%), ammoniumthiocyanate (NH_4_SCN, 97.5%), and methylacetate (MeOAc, >99.5%) were purchased from Sigma–Aldrich. Toluene (≥99.8%) was purchased from VWR Chemicals.

*FAPbI_3_ Thin Film*: Formamidinium iodide (FAI, >98%) was purchased from TCI Chemicals. Lead(II) iodide (PbI_2_, 99.999%), *N*,*N*‐dimethylformamide (DMF, 99.8%), dimethyl sulfoxide (DMSO, ≥99.9%), and chlorobenzene (99.8%) were purchased from Sigma–Aldrich.

*CsSnBr_3_ Thin Film*: Tin(II) bromide (SnBr_2_, 99.2%) was purchased from Alfa Aesar. Cesium bromide (CsBr, 99.9%), *N*,*N*‐dimethylformamide (DMF, 99.8%), dimethyl sulfoxide (DMSO, ≥99.9%), and chlorobenzene (99.8%) were purchased from Sigma–Aldrich.

*MASnI_3_ Thin Film*: Tin(II) iodide (SnI_2_, 99.999%) mesh beads were purchased from Thermo Fisher Scientific. Methylammonium iodide (MAI) was purchased from Solaronix. N,N‐dimethylformamide (DMF, 99.8%), dimethyl sulfoxide (DMSO, ≥99.9%), and chlorobenzene (99.8%) were purchased from Sigma–Aldrich. Tin powder (Sn, 99%) was purchased from Sigma–Aldrich.

*MAPbI_3_ Thin Film*: Lead(II) iodide (PbI_2_, 99.999%) was purchased from Sigma–Aldrich. Methylammonium iodide (MAI) was purchased from Solaronix. N,N‐dimethylformamide (DMF, 99.8%), dimethyl sulfoxide (DMSO, ≥99.9%), and chlorobenzene (99.8%) were purchased from Sigma–Aldrich.

*FASnI_3_ Thin Film*: Formamidinium iodide (FAI, >98%) was purchased from TCI Chemicals. Tin(II) iodide (SnI_2_, 99.999%) mesh beads were purchased from Thermo Fisher Scientific. N,N‐Dimethylformamide (DMF, 99.8%), dimethyl sulfoxide (DMSO, ≥99.9%), and chlorobenzene (99.8%) were purchased from Sigma–Aldrich. Tin pellets (Sn, ≥99.998%) were purchased from Kurt J. Lesker Company.

*FASnBr_3_ Thin Film*: Tin(II) bromide (SnBr_2_, 99.2%) was purchased from Alfa Aesar. Formamidinium bromide (FABr, >98%), N,N‐dimethylformamide (DMF, 99.8%), dimethyl sulfoxide (DMSO, ≥99.9%), and chlorobenzene (99.8%) were purchased from Sigma–Aldrich. Tin powder (Sn, 99%) was purchased from Sigma–Aldrich.

*Self Assembly*: octadecene (ODE, 90%), oleyl amine (OAm, 90%), and oleic acid (OA, 90%) are purchased from Sigma–Aldrich.

All chemicals were used without further purification. All perovskite precursors are freshly purchased and opened inside a nitrogen‐filled glovebox to avoid oxidation or contamination.

### Nanocube Synthesis and Self‐Assembly

Nanocubes are synthesized via the hot injection method as reported before,^[^ ^31^, ^51^ ^]^ followed by self‐assembly to make acceptor layers. Briefly, 2.5 mL OA, 40 mL ODE, and 0.814 g of Cs_2_CO_3_ were loaded into a 100 mL three‐necked round bottom flask to react for 2 h at 150 °C. Then the temperature was changed to 100 °C, following by continuously stirring before use. Meanwhile, a second 100 mL three‐necked round bottom flask containing 0.69 g of PbBr_2_, 5 mL of OA, 5 mL of OAm, and 30 mL ODE were heated to 120 °C to react. Then the temperature was raised to 160 °C. When the temperature reached 160 °C, this solution was injected into the cesium oleate solution prepared above. Immediately after, the flask was put into an ice bath to cool it to about 16 °C. The nanocube solution was then centrifuged two times to purify, then dispersed in toluene.

To perform close packed self‐assembly, first remove the long ligands by adding methyl acetate, followed by adding NH_4_SCN to anchor short ligand SCN− to the nanocubes. 17.4 µL of solution containing nanocubes with short ligands was then put on a 10×10 mm Si or FTO coated glass substrate pre‐immersed in toluene, and placed into a closed glass chamber. A small beaker filled with toluene was then placed next to the substrate to control the toluene vapor pressure inside the chamber. After toluene evaporates overnight, a well packed nanocube layer with (100) orientation was formed and ready to be used in contact transfer epitaxy as the acceptor film.

### Donor Film Preparation

Perovskite precursor solutions were fabricated in two steps. First, the BX_2_ precursors were dissolved in a DMF/DMSO (4:1) solvent mixture. For FAPbI_3_ and MAPbI_3_, a PbI_2_ solution with a nominal concentration of 1.5 m was synthesized, while for FASnI_3_, MASnI_3_, FASnBr_3_, and CsSnBr_3_, SnI_2_ and SnBr_2_ solutions with nominal concentration of 1.0 m were synthesized.

These initial precursor solutions were stirred overnight at 75 °C and 420 rpm. Secondly, the full perovskite precursor solution was fabricated. For FAPbI_3_ and MAPbI_3_, FAI and MAI were dissolved in a PbI_2_ solution with a nominal concentration of 1.24 m. For FASnI_3_, MASnI_3_, FASnBr_3_, and CsSnBr_3_, FAI, MAI, FABr and CsBr were dissolved in SnI_2_ and SnBr_2_ solutions, respectively, with nominal concentration 0.8 m. These final precursor solutions were stirred at 75 °C and 420 rpm for 2 h.

Then the final solutions are stirred for 2 h before use. Sn perovskite solution is known to oxidize quickly even when the solution is stored in a nitrogen‐filled glovebox.^[^ ^52^ ^]^ To prevent Sn^2+^ from oxidizing to Sn^4+^, 1 g Sn pellets or 0.05 g Sn powder were added to Sn perovskite solutions.^[^ ^53^ ^]^ The clean Si, FTO, or ITO substrates were first treated for 45 min in a UV‐ozone cleaner to activate the surface. Then 80 µL perovskite solution was placed onto the substrates, followed by spinning at 3000 RPM for 30s. After 10 s, 200 µL of chlorobenzene was added to the substrates as an anti‐solvent. The substrates were then annealed at 100 °C for 45 min on a hotplate to form perovskite films.

### Contact Transfer

The contact transfer is performed in a tube furnace to achieve a well controlled temperature together with nitrogen protection. Experiments performed using a hotplate in air rather than a tube furnace provided similar but less reproducible results. First the samples were loaded into the chemical vapor deposition (CVD) tube inside a glovebox to be sure the tube was filled with nitrogen. Then the tube on one side was closed and a pressure regulator was put on another side to maintain a stable pressure during heating. The closed tube then was transferred to a pre‐heated tube furnace to perform contact transfer. After that, the samples were moved back to the glovebox to be unloaded. For CsSnBr_3_ perovskites, a layer of poly(methyl methacrylate) (PMMA) dissolved in chlorobenzene was spin coated on top of the films as encapsulation to protect them from air before EDS, XRD, absorption and PL measurements.

*X‐Ray Diffraction*: A Bruker D2 Phaser equipped with a Cu X‐ray source (κα1 with λ = 1.5406 °A, κα2 with λ = 1.5444 °A) was used to obtain X‐ray Diffraction (XRD) patterns.

*Absorbance*: Steady state absorbance spectra were obtained with a Perkin Elmer LAMBDA 750 UV/VIS/NIR Spectrophotometer equipped with a deuterium lamp, tungsten lamp, an InGaAs detector, and an integrating sphere. Samples are illuminated with single wavelength light making use of a monochromator.

*Photoluminescence Spectroscopy*: Photoluminescence (PL) spectra are obtained using a WiTEC Alpha300 SR confocal microscope coupled to a UHTC 300VIS WiTEC spectrometer and a ThorLabs S1FC405 405 nm laser.

*Electron Microscopy Imaging*: Scanning electron microscopy (SEM) images were obtained using an ThermoFischer Verios 460 scanning electron microscope. Energy dispersive X‐ray spectroscopy (EDS) measurements were obtained with the same SEM, along with an Oxford Instruments EDS detector. Electron backscatter diffraction (EBSD) was carried out with the same SEM and a customized timestamping CMOS detector.

PLQY measurements were taken on a home‐built optics set‐up with a coupled supercontinuum laser, integrating sphere, a beam monitor, reflection and transmission photodetector, NIR objective and a 3D piezo stage mounted in the integrating sphere. The system configuration can be found in previous work.^[^ ^54^ ^]^ PLQY was measured with an incident laser set to 532 nm with a power of around 1500 Suns equivalent. iV_OC_ measurements were taken in the same setup, based on a method described in previous work.^[^ ^44^ ^]^

## Conflict of Interest

The authors declare no conflict of interest.

## Supporting information



Supporting Information

## Data Availability

The data that support the findings of this study are available from the corresponding author upon reasonable request.
