# Supplementary material for: Contact Transfer Epitaxy of Halide Perovskites
Source: Adv Mater. 2025 Jul 9;37(39):2308892. doi: 10.1002/adma.202308892 (PMC12506600; doi:10.1002/adma.202308892)
Supplement: Supplementary file 1 — Supporting Information [file ADMA-37-2308892-s001.docx]

**Supplementary**


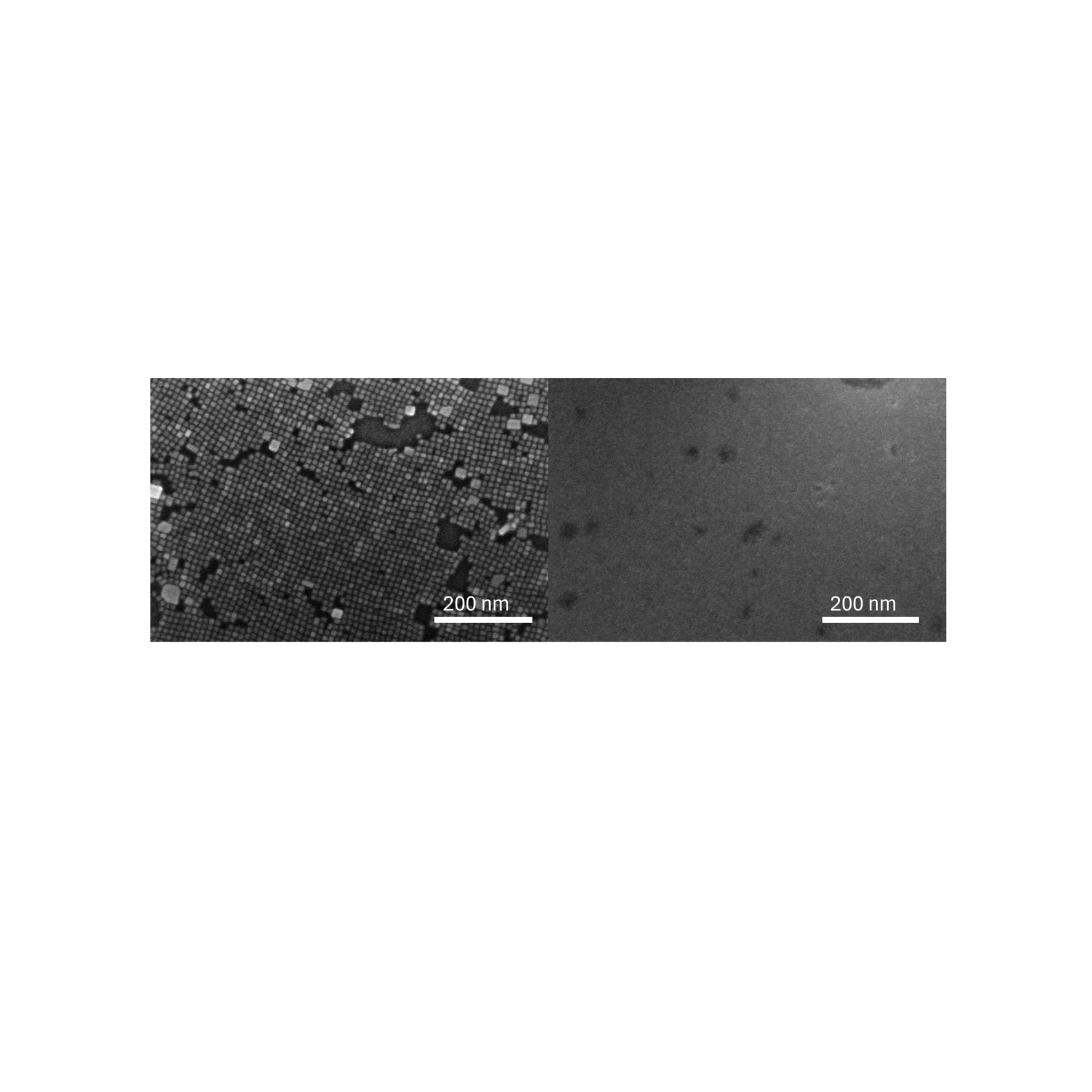


**Figure S1.** The acceptor substrate after the contact transfer process (a) without donor film (b) without nanocube layer (using FAPbI_3_ as donor layer)


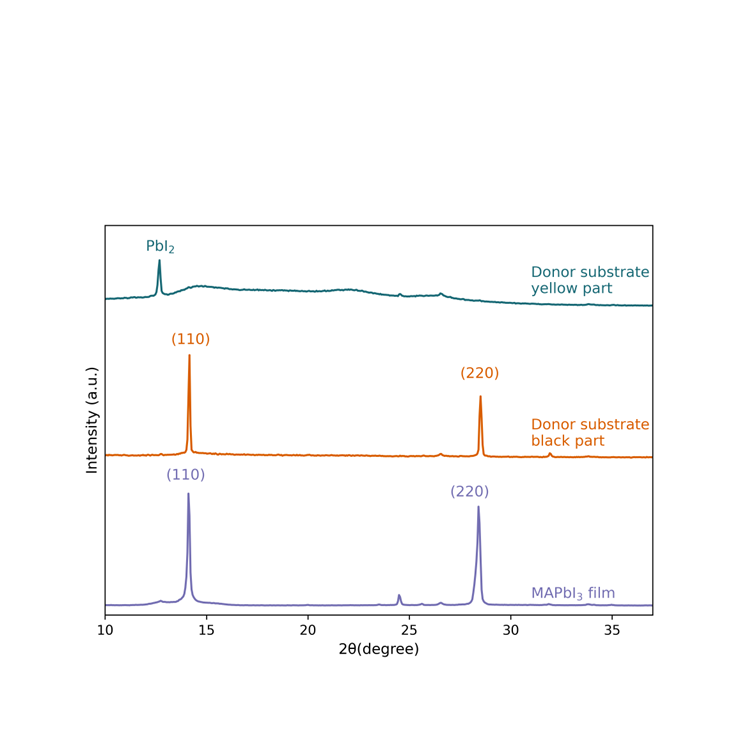


**Figure S2.** XRD pattern of yellow part of the donor substrate after contact transfer, the black part of the donor substrate after contact transfer and of a MAPbI3 film


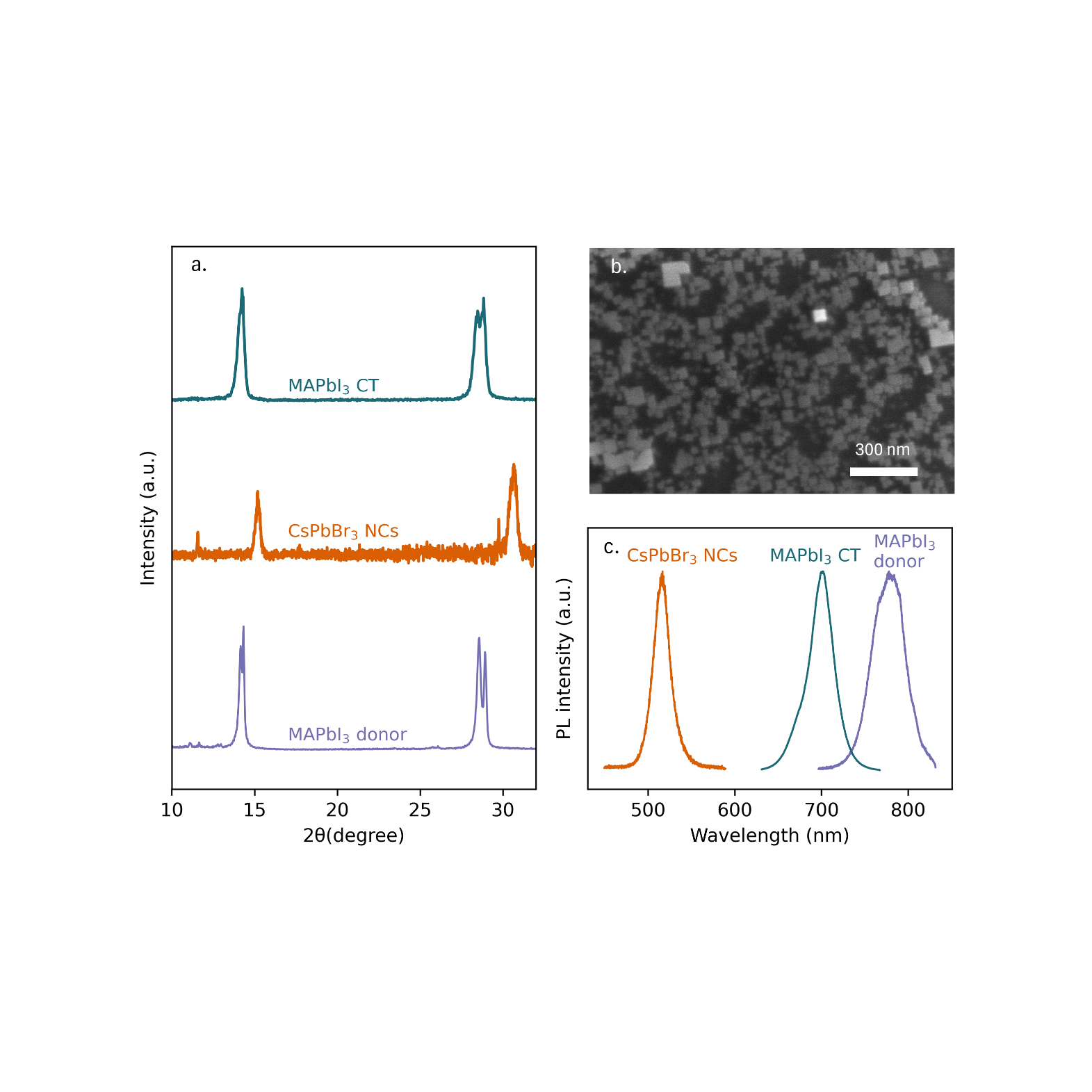

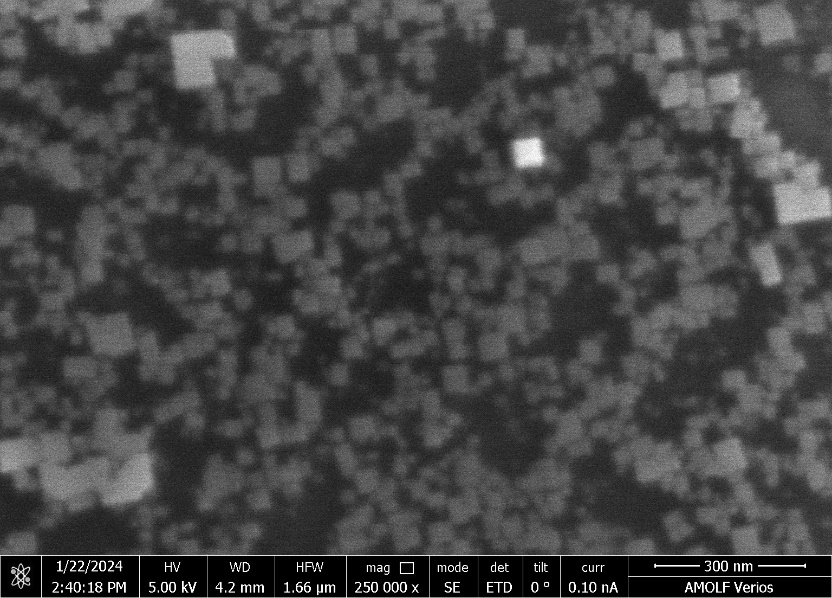


**Figure S3.** Contact transfer of MAPbI_3_. (a) XRD patterns, (b) SEM of MAPbI_3_ contact transfer, (c) PL spectra


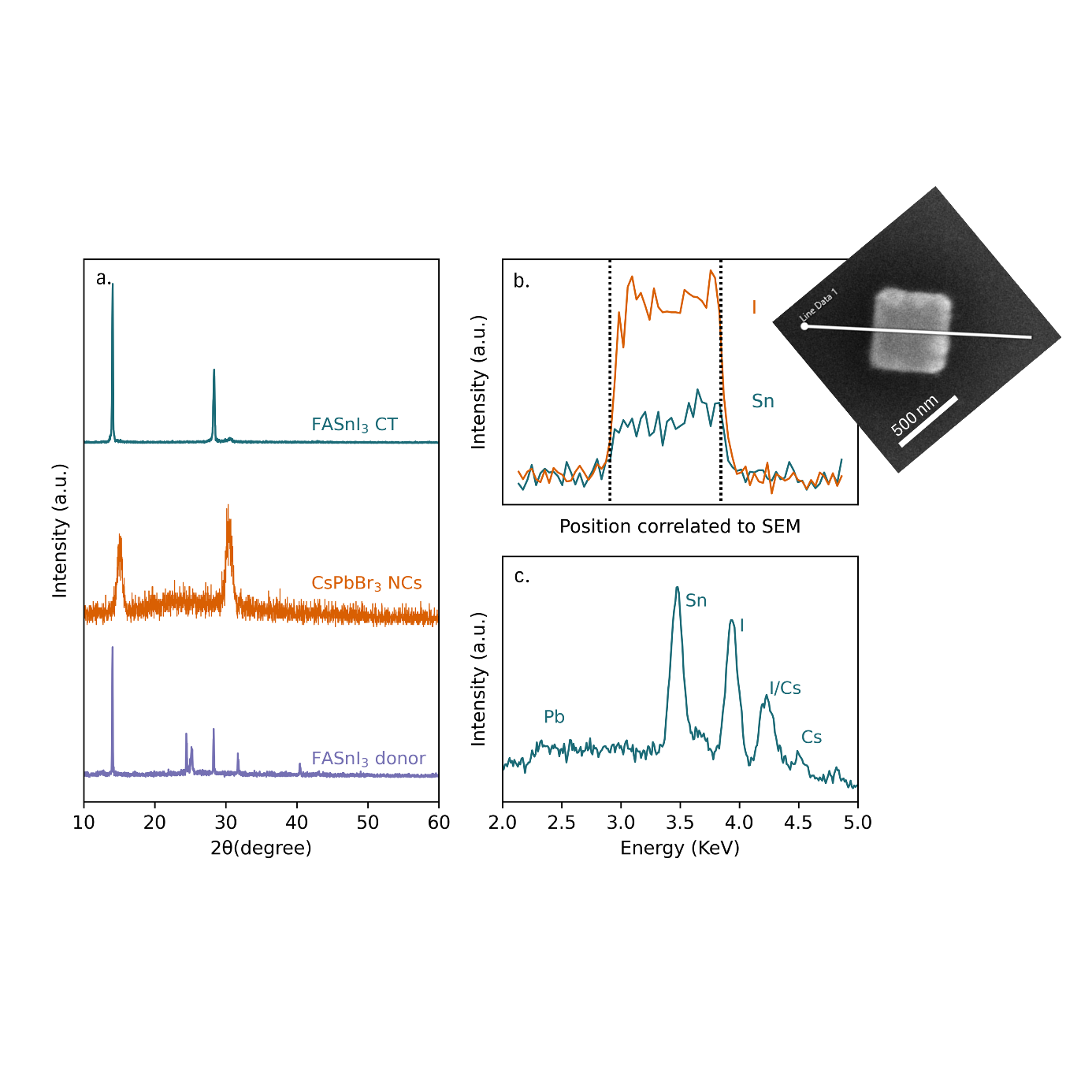

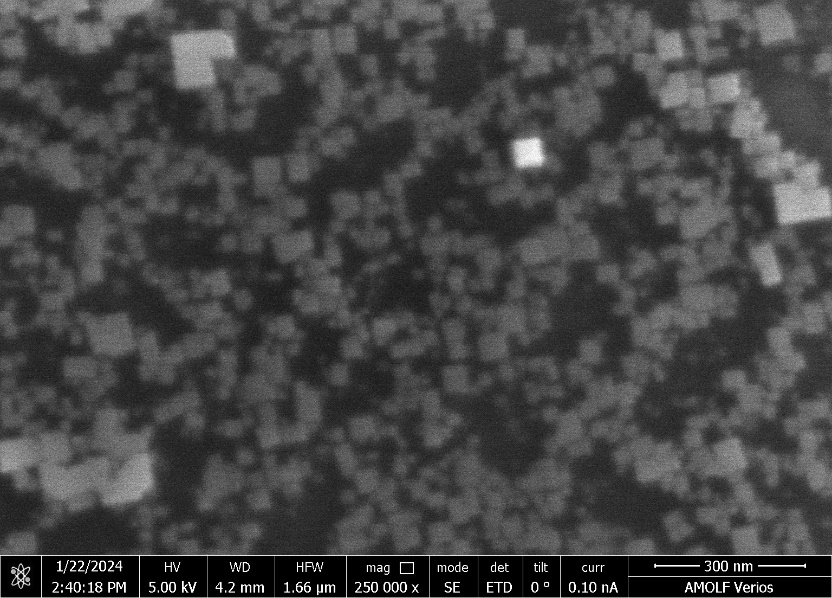
**Figure S4.** Contact transfer of FASnI_3_. (a) XRD patterns, (b) EDS linescan of FASnI_3_ contact transfer, (c) EDS spectrum of FASnI_3_ contact transfer


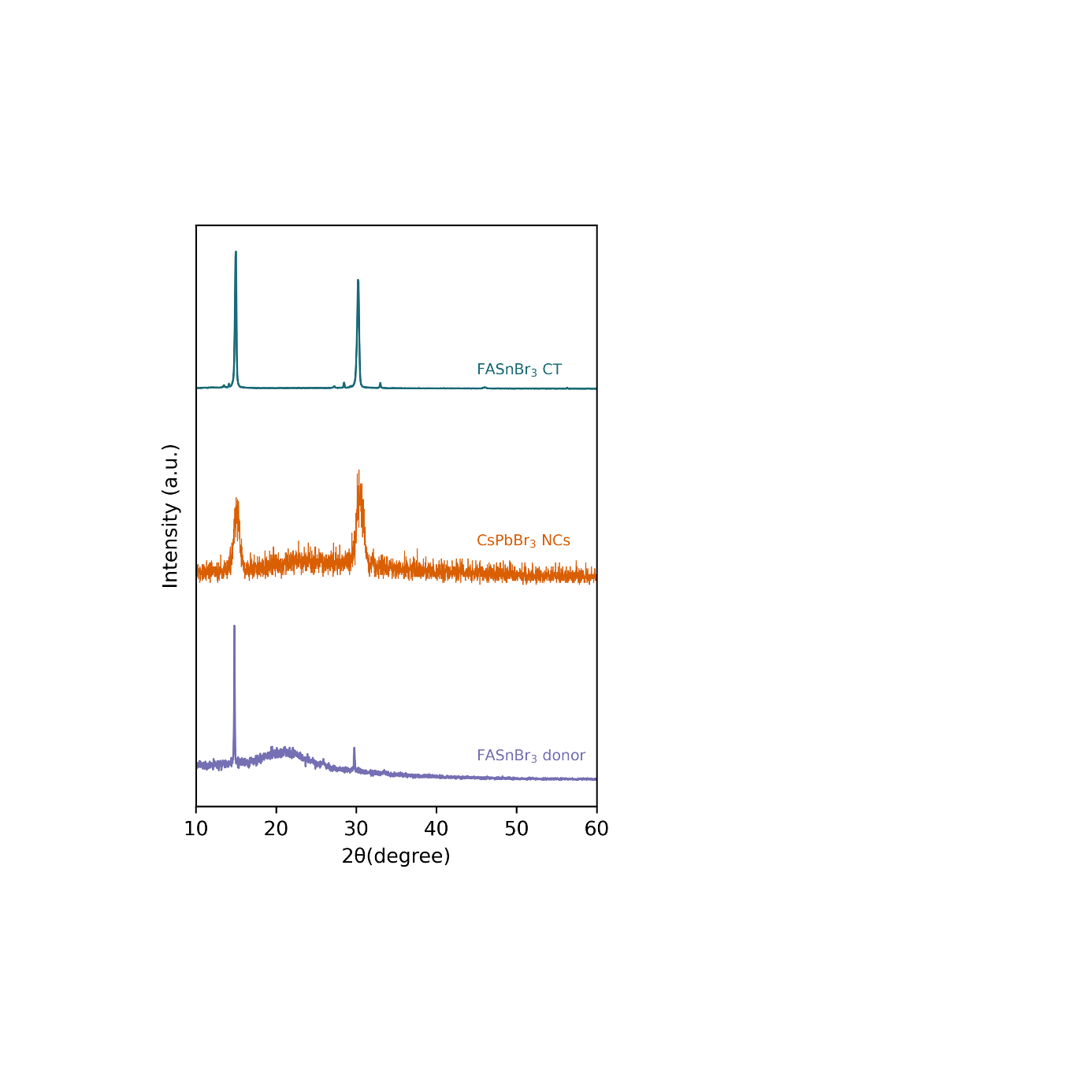


**Figure S5.** XRD pattern of contact transfer of FASnBr_3_


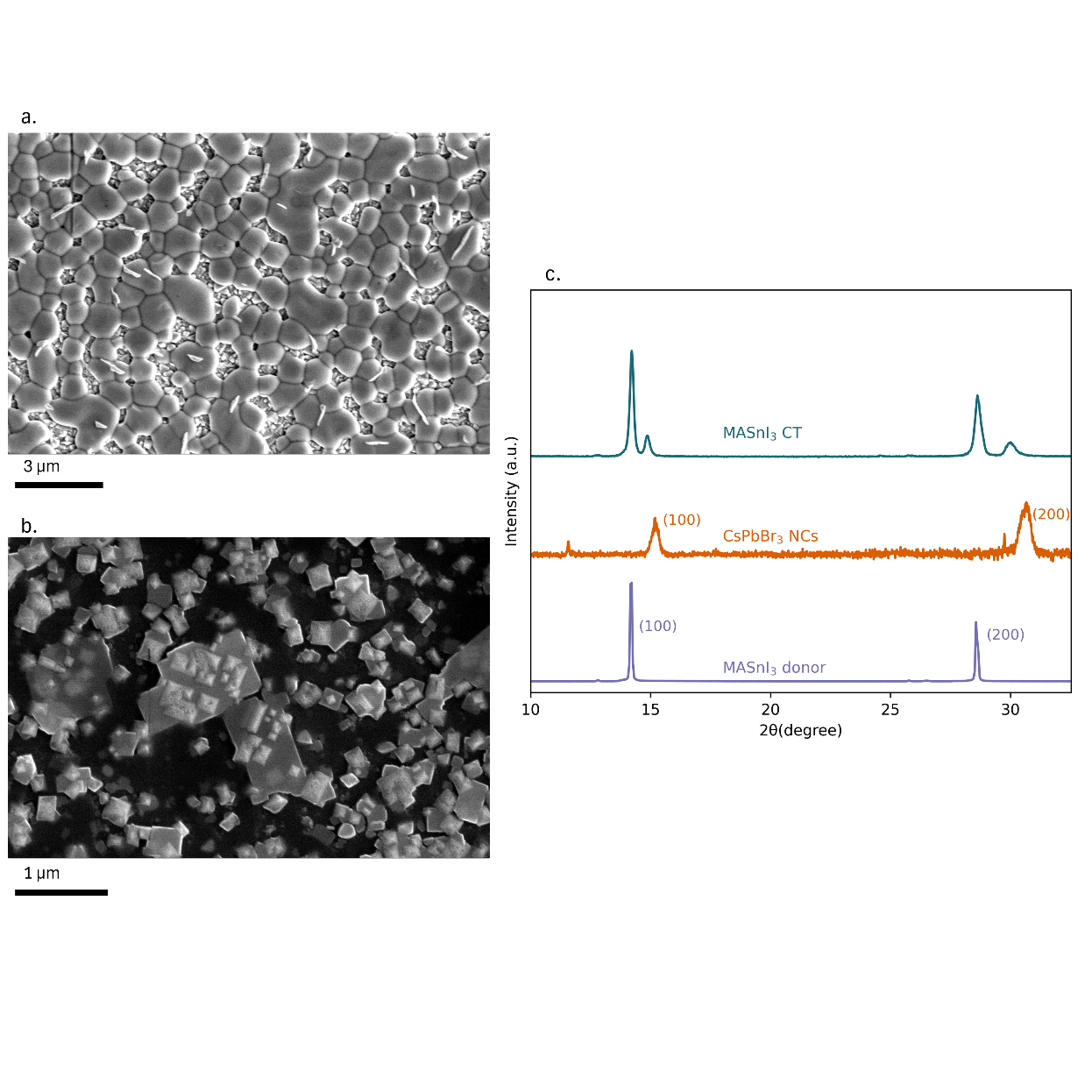


**Figure S6.** Contact transfer on MASnI_3_ at 220°C. (a) Donor film after contact transfer, outside of region of contact transfer, and (b) contact transfer film. (c) XRD patterns of all films


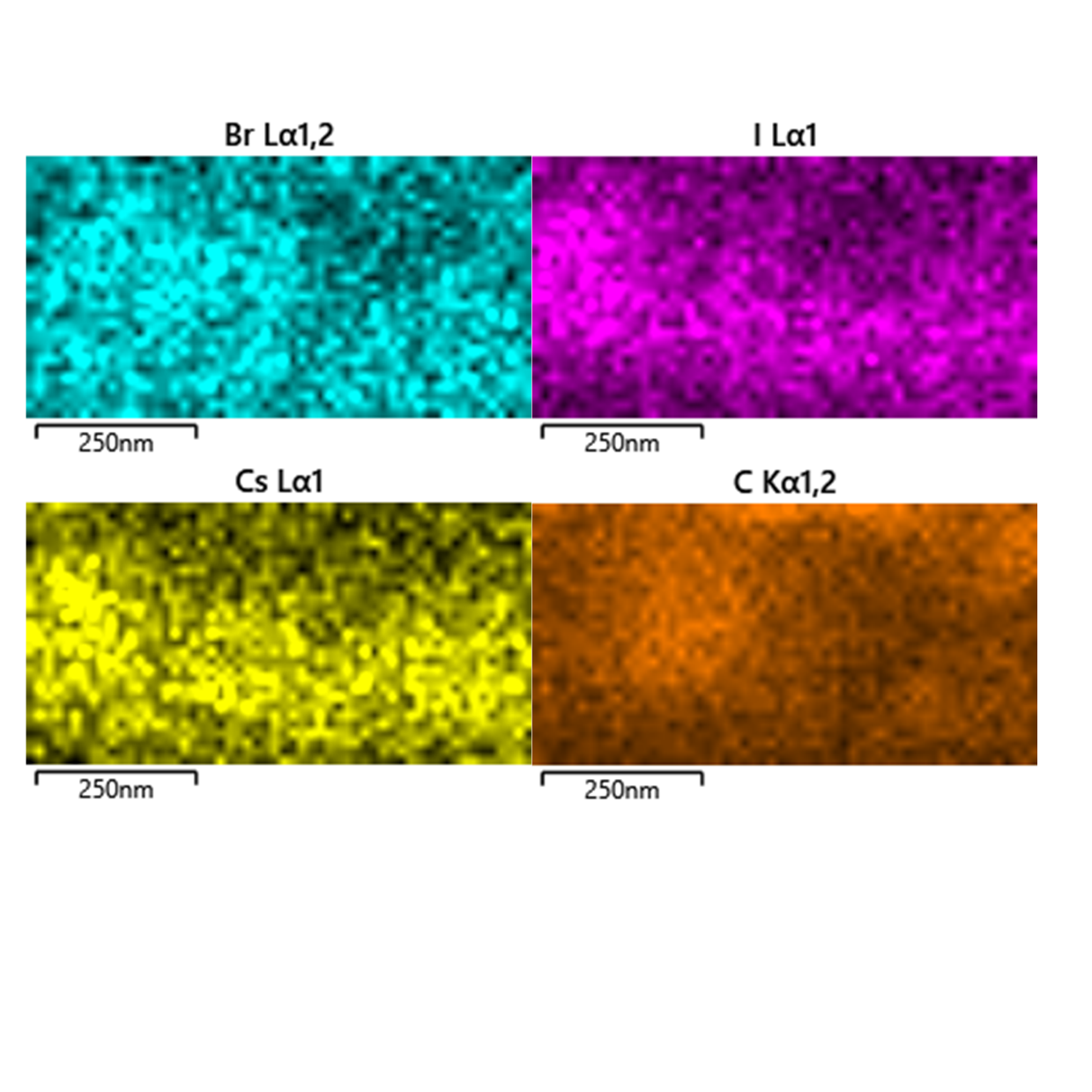


**Figure S7**. Cross sectional EDS map from MASnI_3_ contact transfer


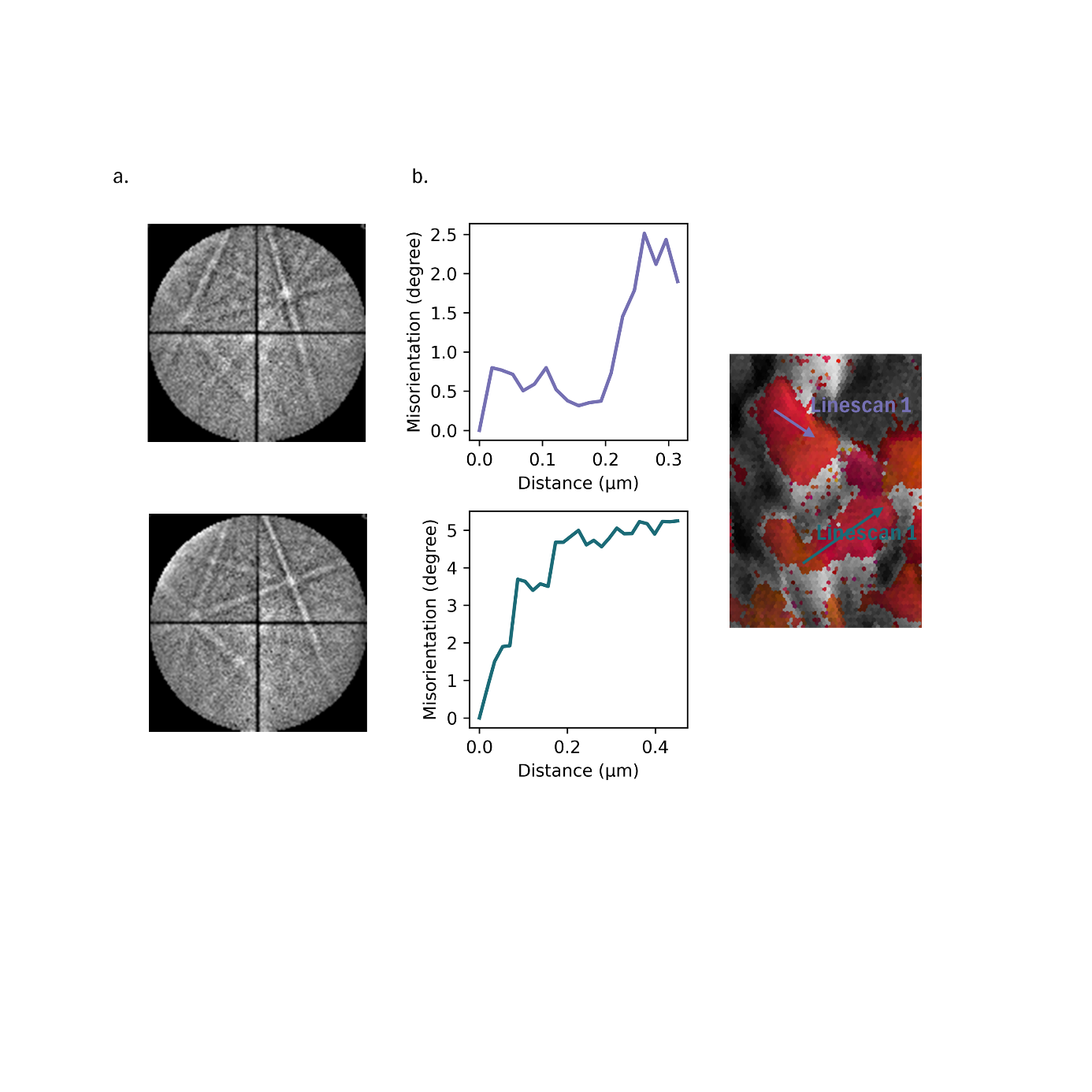


**Figure S8.** (a) Two examples of Kikuchi patterns from the EBSD map in **Figure 5a**. (b) Misorientation from EBSC linescan 1 and 2


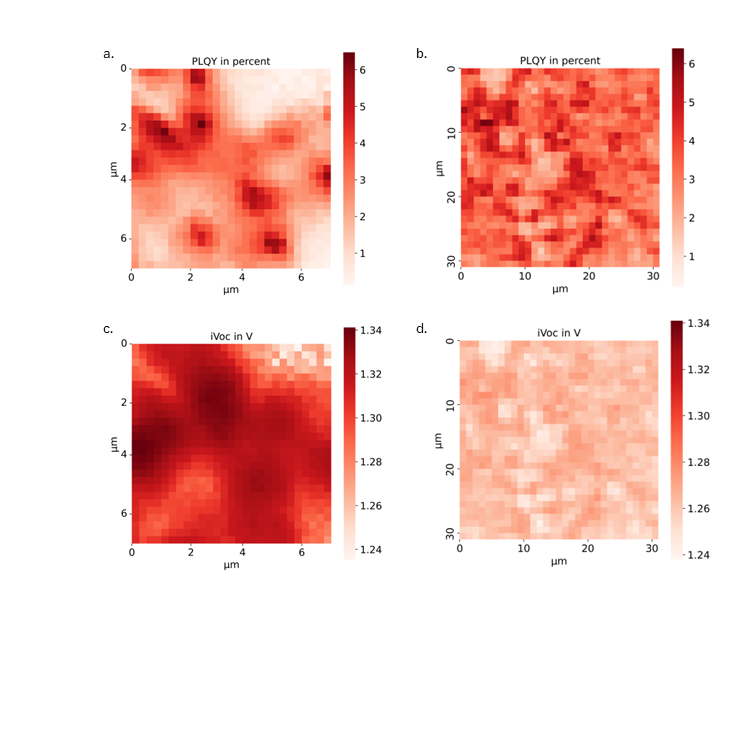


**Figure S9.** Spatially resolved PLQY map and iV_OC_ map of (a, c) acceptor film after MAPbI_3_ contact transfer, and (b, d) MAPbI_3_ donor film before contact transfer
